# Ultra-Thin Plastic Scintillator-Based Proton Detector for Timing Applications

**DOI:** 10.3390/s25030971

**Published:** 2025-02-06

**Authors:** Mauricio Rodríguez Ramos, Javier García López, Michael Seimetz, Jessica Juan Morales, Carmen Torres Muñoz, María del Carmen Jiménez Ramos

**Affiliations:** 1 Centro Nacional de Aceleradores (U. Sevilla, CSIC, J. de Andalucia), 41092 Seville, Spain; fjgl@us.es (J.G.L.); ctorres1@us.es (C.T.M.); mcyjr@us.es (M.d.C.J.R.); 2 Departamento de Física Atómica, Molecular y Nuclear, Universidad de Sevilla, 41012 Seville, Spain; 3 Instituto de Instrumentación para Imagen Molecular (i3M), CSIC-Universitat Politècnica de València, 46022 Valencia, Spain; mseimetz@i3m.upv.es (M.S.); jjuan@i3m.upv.es (J.J.M.); 4 Departamento de Física Aplicada II, Universidad de Sevilla, 41012 Seville, Spain

**Keywords:** thin plastic scintillator, time-of-flight, timing applications

## Abstract

The development of advanced detection systems for charged particles in laser-based accelerators and the need for precise time of flight measurements have led to the creation of detectors using ultra-thin plastic scintillators, indicating their use as transmission detectors with low energy loss and minimal dispersion for protons around a few MeV. This study introduces a new detection system designed by the Institute for Instrumentation in Molecular Imaging for time of flight and timing applications at the National Accelerator Center in Seville. The system includes an ultra-thin EJ-214 plastic scintillator coupled with a photomultiplier tube and shielded by aluminized mylar sheets. The prototype installation as an external trigger system at the ion beam nuclear microprobe of the aforementioned facility, along with its temporal performance and ion transmission, was thoroughly characterized. Additionally, the scintillator thickness and uniformity were analyzed using Rutherford backscattering spectrometry. Results showed that the experimental thickness of the EJ-214 sheet differs by approximately 46% from the supplier specifications. The detector response to MeV protons demonstrates a strong dependence on the impact position but remains mostly linear with the applied working bias. Finally, single ion detection was successfully achieved, demonstrating the applicability of this new system as a diagnostic tool.

## 1. Introduction

Radiation detectors play a crucial role in a wide range of scientific and technological domains, including particle physics, nuclear security, and medical diagnostics [1,2]. Recent advancements in particle accelerators have led to the development of sophisticated detection systems capable of measuring extremely brief time intervals with high temporal resolution [3]. These measurements are particularly crucial for experiments involving the determination of mean lifetimes of excited states, advanced detection systems for charged particles in laser-based accelerators with exceedingly high counting rates [4], and time-of-flight studies (ToF) for precise timing measurements. To meet these requirements, a range of nuclear radiation detectors are employed, including organic and inorganic scintillation counters [5], Cherenkov detectors [6], and semiconductor detectors [7]. Among these technologies, plastic scintillators have emerged as essential sensors due to their unique characteristics and timing properties, including high scintillation efficiency [8], fast temporal response [9], versatility in fabrication, and low cost with respect to conventional crystal scintillators, which facilitate their application in numerous experimental contexts [10].

Plastic scintillators are typically composed of a polymer matrix, often polyvinyl toluene (PVT), doped with organic compounds. These scintillators emit light due to the ionoluminescence process, a phenomenon in which ionizing radiation excites the doped organic compounds, leading to the emission of optical photons, predominantly in the visible spectrum [11]. This light emission, known as scintillation, is detected by photosensitive devices such as photomultiplier tubes (PMTs) or photodiodes, which enable the quantification and analysis of the incident radiation [12]. The versatility of plastic scintillators extends to numerous applications: In high-energy physics [13], they are used as detectors for laser-accelerated protons [14] and calorimetry [15], providing critical data on particle interactions and energy deposition. In medical physics, plastic scintillators are employed in positron emission tomography (PET) [16] and radiation therapy dosimetry [17], where precise and real-time measurement of radiation doses is essential. Additionally, in security and cosmogenic radiation monitoring, these scintillators are utilized for detecting and quantifying radioactive materials, contributing to safety and regulatory compliance [18]. Very thin (<60 μm) plastic scintillators coupled to the surface of position-sensitive photomultiplier tubes have been utilized for the spatially resolved detection of α and β particles [19,20]. These thin-film detectors (TFDs) provide high scintillation light output with minimal energy loss of transient particles, enabling ToF measurements of light ions in low-energy nuclear physics experiments [21,22]. A prominent example of these materials is the commercial ultra-thin plastic scintillator EJ-214. This work focuses on the development and characterization of a new detector based on this novel plastic scintillator designed for particle detection and to be used as an external trigger within the microprobe line at the National Accelerator Center (CNA) in Seville [23]. Utilizing this new system as an external trigger will address the challenges associated with relying on auto-trigger modes during the characterization of the temporal response of nuclear detectors, such as those employed by the Time-Resolved Ion Beam Induced Charge (TRIBIC) technique [24]. Moreover, the enhanced timing capabilities of this new system will support the investigation of temporal processes in our microbeam chamber, including the study of transport properties in semiconductor detectors. This paper is organized as follows: The introduction outlines the advantages of the new thin plastic scintillator detector as a radiation detector and provides an overview of the current state of the art and applications of this type of system. In Section 2, we describe the new detection system, focusing on its design, construction, and installation in the CNA’s nuclear microprobe. Section 3 discusses the thickness and uniformities of the detector, based on the analyses performed using the Rutherford Backscattering Spectrometry (RBS) technique. Section 4 covers the characterization of the prototype performance, including the effects of ion impact location on the detector response, the response as a function of the applied bias, and the feasibility of single ion detection using pulsed proton beams. In the next section, we examine the transmission of ions through the collimator slits, studying the angular divergence of the outgoing ions from the scintillator using the Monte Carlo software SRIM 2013 [25]. The paper continues with presents an overview of the experimental setup and the application of the new detector for ToF measurements. Finally, the conclusions summarize the key findings related to the performance of this detection system.

## 2. Materials and Methods

The developed detection system utilizes a commercial ultra-thin organic scintillator material, Eljen EJ-214, which is manufactured by Eljen Technology (Sweetwater, TX, USA) [26]. This material is specifically formulated for use in ultra-thin films designed for heavy ion studies and beam monitors [27]. EJ-214 emits light in the blue region of the visible spectrum, with maximum emission centered at 435 nm. One of the main advantages of EJ-214 is its fast decay time, in the order of nanoseconds, which allows for rapid detection and excellent temporal resolution of fast events. This makes EJ-214 scintillators ideal for applications requiring high-speed measurements, such as triggering.

Additionally, the light efficiency and good transparency of EJ-214 enhance its effectiveness in various detection scenarios. This improvement is attributed to the very high concentration of wavelength shifter dopants, which enhance the matching between emission and absorption, thereby shifting the primary scintillation light. This optimization increases the light emission for particle beam timing applications while minimizing beam energy deposition. Table 1 summarizes the main properties of EJ-214 according to the supplier. Based on these properties, a timing detector concept using this ultra-thin plastic scintillator was developed and constructed by the Institute of Instrumentation for Molecular Imaging (I3M). While detectors using thicker scintillators are documented in the literature [28], the major innovation of this approach is the ultra-thin scintillator, with a nominal thickness of 25 μm, as specified by the supplier.

The thickness of this new system enables the detector to be applied in transmission mode, as ions with energies in the range of a few MeV can pass through the active volume of the detector without losing a significant amount of energy. For efficient scintillation light collection, two approaches have been historically proposed: either by reflecting it off an external hollow mirror or by using an optically transparent guide that covers a significant portion of the TFDs’ surface [29,30]. This capability facilitates ToF and timing measurements. During the construction, a single portion of an EJ-214 scintillator sheet (active volume) with dimensions 10 × 45 mm was placed between two polymethylmethacrylate (PMMA) semi-cylinders (manufactured by Kümpel [31]) in a sandwich structure, introduced ≈10 mm from the edge. The dimensions of the PMMA cylinders are 10 mm in diameter and 30 mm in height, and the dimensions of the scintillator sheet are assumed to be 10 × 45 × 0.025 mm. As ionizing radiation passes through the material, a light flash is generated with a decay time of approximately 2 ns. These photons are directed by the PMMA cylinders, which match the scintillator geometry and act as light guides, transporting the photons to a commercial photomultiplier tube (model R647 with an E849-35 socket by Hamamatsu (Shizuoka, Japan) [32]) with a 10 mm diameter photocathode, maximum quantum efficiency at 420 nm, and a rise time of 2.1 ns according to the datasheet. The other portion of the active volume is completely covered with a double layer of aluminized mylar on both sides, shielding the assembly from the background light. After completing the assembly, the main components are encased in a plastic housing manufactured via 3D printing.

**Figure 1:**
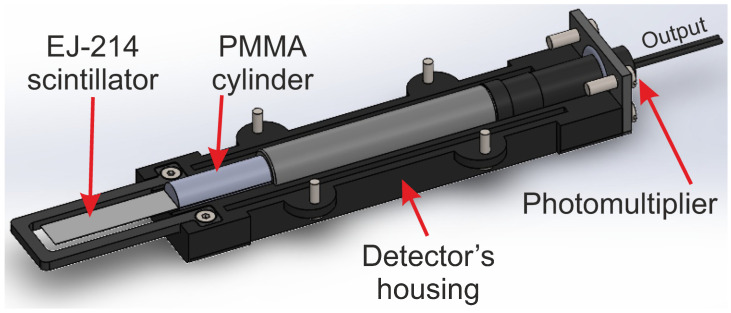
Cross-section of the basic design of the plastic detector, illustrating the main components housed within a plastic casing manufactured using 3D printing.

The maximum dimensions of the casing, including screws, are 194 mm (length) × 46 mm (width) × 32 mm (height). The output signal is an analog pulse from the PMT anode. Two cables emerge from the PMT socket: one for the high voltage power supply and the other for the anode voltage (output signal). A schematic of the detector layout, showing the main components, is depicted in Figure 1.

**Figure 2:**
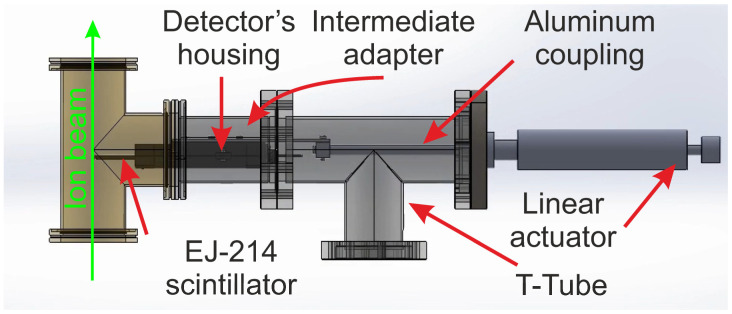
Diagram of the auxiliary mechanical system showing the main components for the assembly in the nuclear microprobe beam line.

One of the primary applications of this new type of detection system is to be used as an external trigger or for ToF measurements in facilities that work with ion beams, such as the ion beam nuclear microprobe at CNA. For this purpose, the detector module was designed with several mechanical components to ensure its effective integration and operation within the beamline, as well as its ability to intercept the ion beams. It must be securely attached under vacuum conditions, and the system must facilitate the movement of the detector between positions within and outside the ion beam trajectory without disrupting this vacuum. The current assembly at CNA was installed before the collimator slits, at a distance of 38 cm, as illustrated in Figure 2.

This assembly includes a tube, labeled T, which is a component of the tandem beamline in the accelerator. The new assembly has been attached to the side output of this tube. To allow the detector to move between positions inside and outside the ion beam, a 100 mm linear actuator is employed. The actuator is mounted at one end of the T-shaped tube, which also requires an additional reducer for secure attachment. The other end of the tube connects to the beamline via an intermediate adapter. The side output of the T-shaped tube is sealed with a flange that incorporates two BNC bulkhead connectors: one for the anode readout and the other for the PMT polarization. In this setup, the actuator features a cylindrical rod that moves back and forth.

An additional aluminum coupling securely holds the detector and supports its weight. The main module is attached to the front bar of the actuator. When the actuator is extended to its full length, the detector is fully positioned in the ion beam path, ensuring precise alignment and effective operation within the particle accelerator system. All major components of the proposed assembly were supplied and manufactured by JEV Instruments [33].

## 3. Results and Discussion

### 3.1. Thickness Characterization Using RBS

**Figure 3:**
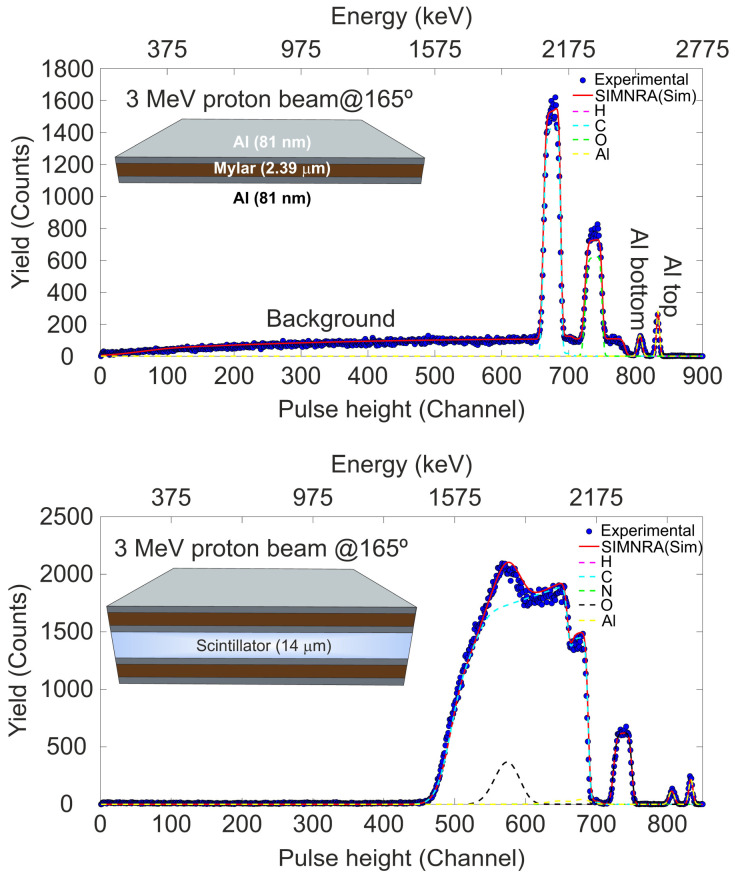
Experimental and simulated RBS spectra obtained with a 3 MeV proton beam. The top panel shows data for a piece of aluminized mylar, while the bottom panel presents data for the sandwich structure containing the aluminized mylar shield over an EJ-214 scintillator sheet.

A comprehensive understanding of the structure and composition of the elements present in the new system based on the plastic detector is essential for correctly interpreting the obtained results during its operation, both in terms of its response and its use as a ToF detector. The thickness of the main components of the detector was determined using the RBS technique [34]. For the analyses, the sandwich structure that forms the final detector, consisting of aluminized mylar/scintillator/aluminized mylar, was assembled. To facilitate the analysis of the spectra, one of the mylar sheets was also measured separately. In this study, RBS measurements were conducted using an IBM geometry in the multipurpose chamber of the 3 MV Tandem accelerator of the CNA. The samples were installed in a rectangular sample holder (150 × 112 mm^2^) with holes (to avoid its signal in the spectra), which can be moved in the X and Y directions (perpendicular to the direction of the incident ion beam) using stepper motors. The analysis was carried out using protons accelerated to an energy of 3 MeV with a beam current of 1 nA. The RBS spectra were measured with a solid-state detector (passivated implanted planar silicon detector or PIPS) with an active area of 50 mm^2^, positioned at 165°. The beam diameter was set to 1 mm, and measurements were made every 5 mm to check the homogeneity of the plastic scintillator. The data acquisition (DAQ) system for the RBS measurements in the multipurpose chamber is described in [35]. The energy calibration of the DAQ system was performed using a sample consisting of a thin layer of Au deposited on a C substrate, together with the spectrum of the aluminized mylar target. Figure 3 shows the experimental RBS spectra alongside the simulations obtained using the SIMNRA code [36] for both the aluminized mylar sample and the complete assembly with the EJ-214 sheet.

The inner figures show the layout structure and experimental thickness of the analyzed samples. Both mylar and scintillator contain hydrogen, so it is crucial to remark that in the RBS spectra the H signal does not appear, although it is essential to include it in the simulation to correctly calculate the stopping power. The analytical procedure assumed the nominal composition for these compounds provided by the supplier and adjusted the thickness accordingly. In the case of the aluminized mylar, although a background signal is observed coming from the rear of the vacuum chamber, the structure and thickness of the mylar and the Al coating on both sides are clearly visible. For the simulation of the sandwich structure, the same mylar composition and thickness measured in Figure 3 (Top) were used for both the top and bottom layers, along with the theoretical composition of the EJ-214 scintillator. The free parameters were the thickness and roughness of this material. To achieve a good simulation of the RBS spectrum, it was necessary to include a scintillator roughness with a thickness distribution FWHM of 30,000 × 10^15^ at/cm^2^, approximately 20% of the thickness of the scintillator. Although, at high energy, the Al and O signals from the top mylar layer are clearly separated, the C signal is partially overlapped with the C signal from the scintillator in this case. The thickness determined by RBS (expressed in units of 10^15^ atoms/cm^2^) for the aluminized mylar sheet and the plastic detector are listed in Table 2 and Table 3, respectively. The corresponding physical thickness was calculated based on the theoretical densities of the materials: SRIM provides the density for the aluminized mylar shield, while the density for the scintillator was supplied by the manufacturer (ρ = 1.02 g/cm^3^) [27].

**Figure 4:**
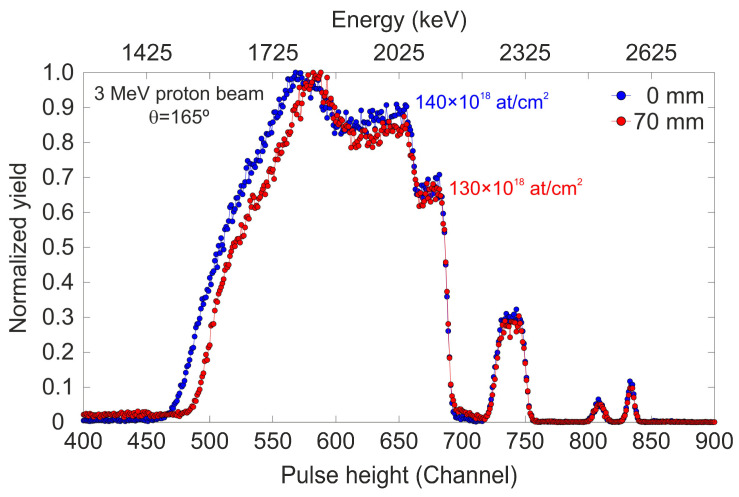
Experimental RBS spectra of the scintillator sheet obtained at positions x = 0 mm (blue) and x = 70 mm (red). These two positions represent the minimum and maximum thickness measured over the scan.

Given that the primary application of this type of thin detector involves ion transmission, ensuring the homogeneity of the plastic sheet thickness is a critical factor. Any significant variation in thickness could influence the energy loss and angular deviation of the ion beams during their use. To evaluate the thickness homogeneity of the EJ-214 sample, a series of RBS measurements were conducted with a 3 MeV proton beam at different positions with 5 mm intervals, covering a total length of 85 mm of the sample. The target was moved using stepper motors, which allowed for precise horizontal and vertical displacement with an accuracy of 10 μm. Figure 4 illustrates the RBS spectra for two positions (0 mm and 70 mm) along the horizontal axis, selected to represent the minimum and maximum thickness measured during the irradiation scan, with all other measurements falling within this range. No spatial dependence on thickness was observed across the measured points between these two values. Based on these results, the experimental thickness varies between 13 and 14 μm with an uncertainty in the adjustment of around 5%, indicating excellent homogeneity in the active volume of the EJ-214 sample. The composition matches the nominal value, but the scintillator thickness is 46% below the supplier average (25 ± 7 μm). The experimental thickness is crucial for accurately calculating the ion energy loss through the detector when conducting simulations with SRIM, which were carried out during the development of this study. Finally, it is important to emphasize that the piece analyzed by RBS is not the same as the one installed in the detector. For the detector assembly, a 10 × 45 mm^2^ piece was cut from the original scintillator sheet (dimensions 100 × 100 mm^2^), in close proximity to the region used for RBS analysis (a piece with dimensions 100 × 20 mm^2^). This precaution was taken because the scintillator sheet was exposed to ion currents on the order of nA, with a beam diameter of 1 mm (current density of 1.27 × 10^−7^ A/cm^2^) during RBS characterization.

Such current densities could potentially degrade the light emission properties of the scintillator. To avoid radiation-induced damage and ensure optimal performance in the detector, the analyzed piece and the one installed in the detector were different.

### 3.2. Variability of Detector Response with Impact Point

Due to the detector dimensions, it was not feasible to perform the characterization as a function of the irradiation location in the ion beam microprobe chamber; for this reason, the detector characterization was conducted at the ion implantation beam line at the CNA [37]. The detector was mounted on an electrically isolated platform inside the vacuum chamber before being irradiated. To determine the position of the ion beam, a high-luminosity scintillator material, SrGa_2_S_4_:Eu^2+^ (commonly referred to as TG-Green [38]), was placed in the same plane and in close proximity to the detector. A high-resolution Charge-Coupled Device camera localized in one external port of the vacuum chamber allows to record the light emitted by the scintillator sample and determine the ion beam position. With the aluminized mylar shielding, ambient light can induce some interference signal in the PMT; so, to ensure the accuracy of the detector response to the ion irradiation, all illumination sources in the vicinity of the vacuum chamber were switched off, thereby achieving complete darkness. A set of collimator slits with a rhombohedral shape was employed along the ion beam line, effectively reducing the ion beam dimensions and decreasing the current intensity over the sample to avoid saturation of the PMT. For these experiments, a pulsed proton beam was employed. To achieve this, we used the beam kicker installed at the output of the ion sources. The kicker consists of a pair of metal plates, to which a high voltage can be applied through a fast solid-state switch, model FSWP 51-02 from Behlke (Dreieich, Germany), in order to deflect the low-energy protons. The bunch length and repetition rate were controlled by an input pulse generator, model AFG 310000 series from Tektronix (Beaverton, OR, USA). The temporal profiles of the pulsed ion beam were recorded using the output signal from the PMT connected to a fast oscilloscope (LeCroy, New York, NY, USA, HDO9404, 4 GHz bandwidth, 40 Gs/s) with 50 Ω input impedance.

**Figure 5:**
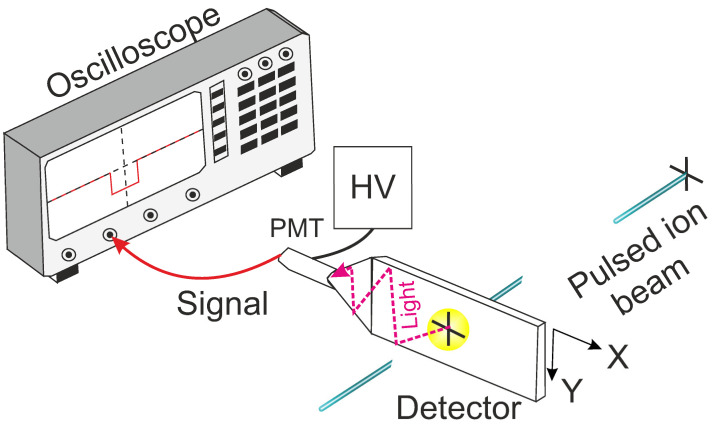
Experimental setup of the implantation chamber. The detector was exposed to a pulsed proton beam and the response was recorded using a fast oscilloscope as a function of the ion beam position.

Finally, a commercial high-voltage power supply (NHR220r HV Source from iseg, Radeberg, Germany) was used to apply the working bias to the PMT. A schematic of the experimental setup is displayed in Figure 5.

**Figure 6:**
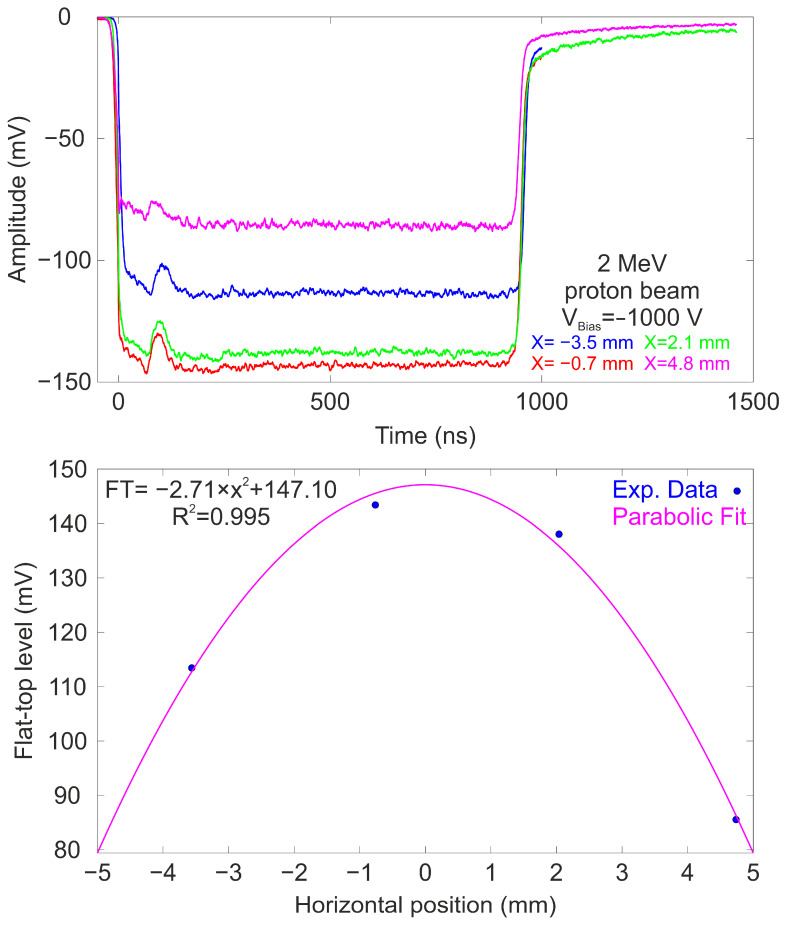
(**Top**) Averaged temporal waveforms of the detector exposed to a 2 MeV pulsed proton beam at different horizontal locations ranging from −3.5 mm to 4.8 mm. The PMT was biased at −1000 V. (**Bottom**) Flat-top level plotted against the impact position along the horizontal axis. The magenta line indicates the best parabolic fit to the set of data.

This set of measurements aims to investigate the dependence of a scintillation detector response on the specific impact points of the ion beam. During the measurements, the ion beam was kept stationary to ensure uniform irradiation, while the target was moved along the X–Y axes using stepper motors that allowed for precise linear displacement along both axes, with a spatial resolution of 0.1 mm per step. This precision was crucial for accurately positioning the ion beam and ensuring that the scans covered the detector area thoroughly. During this characterization a 2 MeV pulsed proton beam was employed. In continuous mode, the beam was configured to have a current of ≈1.6 nA and a beam size of ≈1 mm^2^. The beam current was measured using a current integrator (Model 1000C by Brookhaven Instruments Corporation, New York, NY, USA). To mitigate the risk of saturating the PMT due to excessive light generated in the scintillator, the pulsed beam featured a pulse width of 1 μs and a repetition rate of 1 kHz, delivering approximately 9400 protons per pulse. During the characterization, the detector was biased at −1000 V, and at each designated irradiation point, a minimum of 8000 waveforms were recorded. These data were subsequently processed offline using a custom Matlab script, which averaged the waveforms to enhance the signal-to-noise ratio. For the horizontal scan, which corresponded to the minor dimension of the detector, four distinct impact points were selected at a fixed vertical position of 17.5 mm from the PMMA, with each point spaced 2.8 mm apart. In the vertical direction, representing the major dimension of the detector, eight impact points were chosen, centered at X = 0 mm (the center of the horizontal dimension), with spacing carefully determined to ensure complete coverage of the detector surface, with each point spaced 5 mm apart. Figure 6 (Top) illustrates the temporal response of the detector to the pulsed beam at various horizontal positions along the detector ranging from −3.5 mm to 4.8 mm.

**Figure 7:**
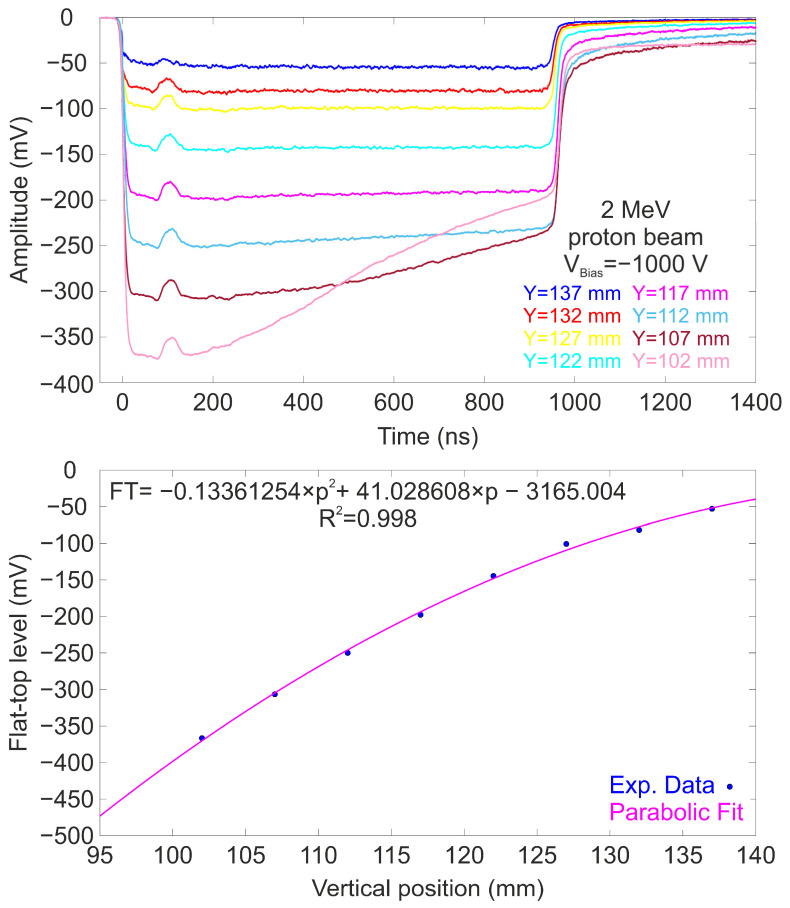
(**Top**) Averaged temporal waveforms of the detector exposed to a 2 MeV pulsed proton beam at different vertical positions ranging from 0 mm to 35 mm. The PMT was biased at −1000 V. (**Bottom**) Flat-top level plotted against the impact position along the vertical axis. The magenta line represents the best parabolic fit to the data set.

All the averaged waveforms display a predominantly flat profile with duration of ≈1 μs and a strong dependence with the impact position. The slight hump observed at the beginning of each signal trace is attributed to an artifact induced by the beam kicker. The bottom panel presents the mean amplitude values (flat-top levels) of the detected signals as a function of the beam position. The experimental data, marked in blue, were fitted to a parabolic function using the least squares method, showing good agreement (R^2^ = 0.995). The same procedure was used to perform a scan along the vertical axis. Figure 7 presents the average waveforms for various positions along the vertical axis.

**Figure 8:**
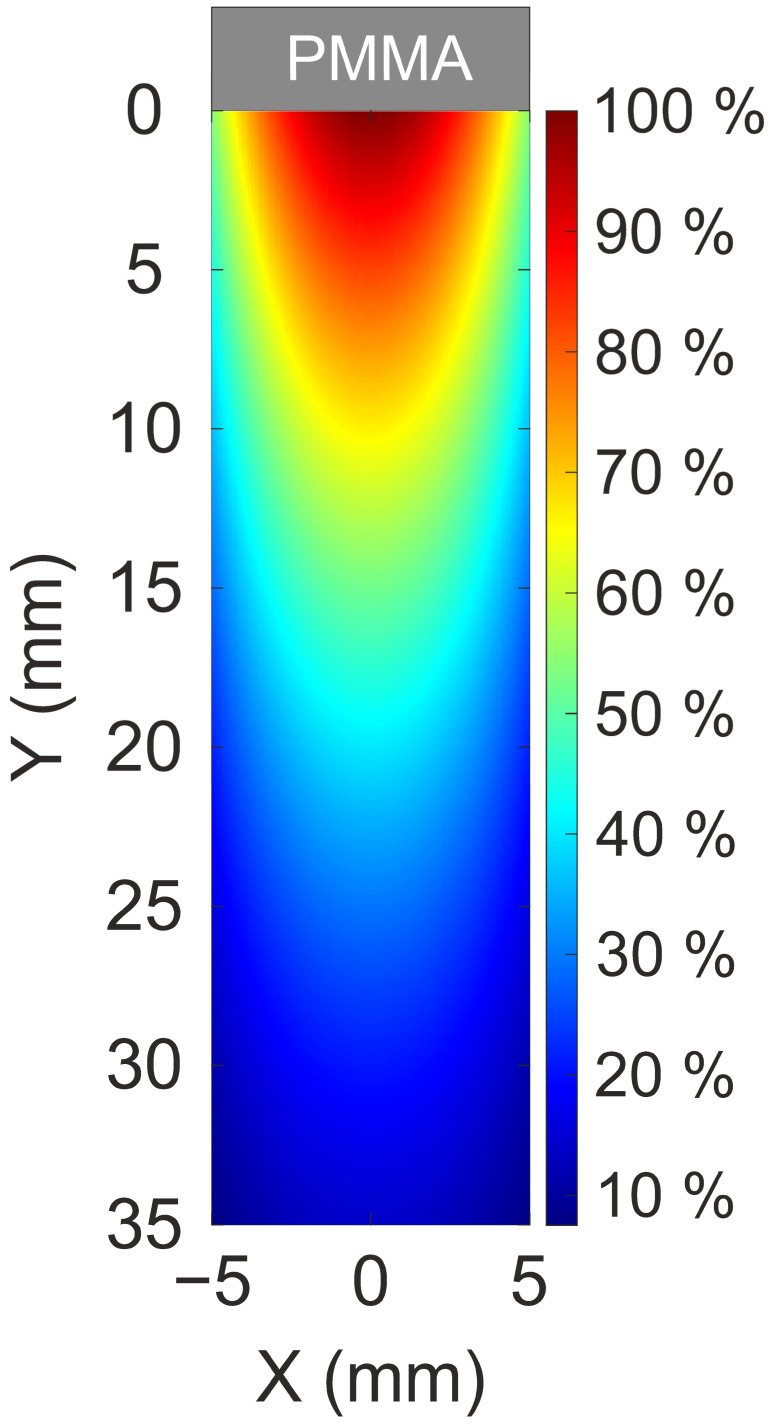
Two-dimensional response map of the detector exposed to a 2 MeV pulsed proton beam, illustrating how the response varies across its surface.

Similar to the horizontal profiles, the flat-top levels of the temporal waveforms are highly dependent on the impact position along the vertical axis. Once again, it is observed that the temporal profiles generally exhibit a flat region, although this is not uniformly observed across all irradiated positions in this case. For amplitudes exceeding 250 mV, a slight slope is noticeable in the temporal profile. This deviation occurs when the amount of light incident on the photocathode exceeds a certain threshold, causing the PMT to experience difficulties in sustaining the signal over extended periods. This phenomenon has been previously observed in similar photocathodes exposed to high illumination levels using calibrated lamps, where deviations from a flat profile were noted [39]. Similarly to the horizontal profile, the experimental data were fitted to a parabolic function, showing very good agreement (R^2^ = 0.998) with the measured values. A two-dimensional response map of the detector was constructed using an interpolation method based on the fitting functions derived from horizontal and vertical scans. The normalized response map I_Norm_(x,y) at any location (x,y) was calculated using the following expression (1):

(1) $$I_{Norm}(x,y)=\frac{\left|I_{x}(x)|\times |I_{y}(y)\right|}{\max\left(\left|I_{x}\right|\right)\times \max(|I_{y}|)}$$

where I_Norm_(x,y) represents the detector response at the location (x,y) in %, I_x_(x) denotes the amplitude at location x obtained from the horizontal parabolic function using linear interpolation, and I_y_(y) denotes the amplitude at location y derived from the vertical parabolic function using the same method. For the application of this 2D model, the origin of the horizontal axis was defined by the vertex of the parabolic fit (x = 0 mm) from the horizontal scan data. For the vertical axis, the origin was set at y = 0 mm, aligning the zero point with the location closest to the PMMA where the amplitude reaches its maximum. Figure 8 illustrates the 2D map of the detector response.

**Figure 9:**
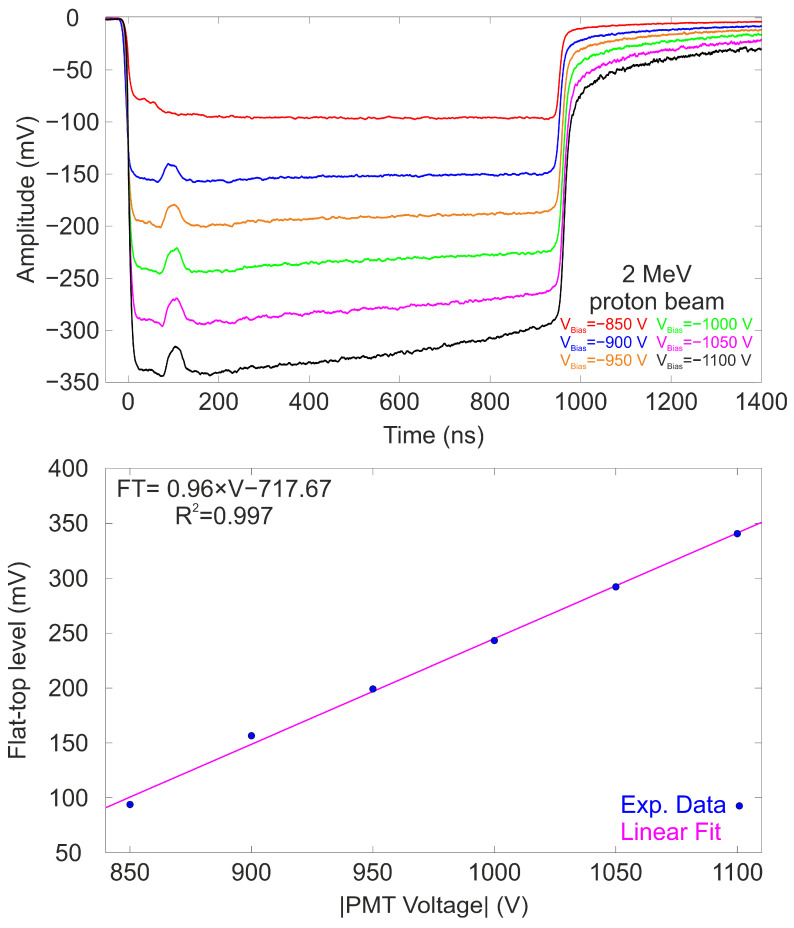
(**Top**) Averaged temporal waveforms of the detector exposed to a 2 MeV proton beam at different biases. (**Bottom**) Flat-top level plotted against the applied bias to the PMT. The magenta line indicates the best linear fit.

The dimensions of the map were 100 × 350 pixels, with each pixel representing a 0.1 mm step. The map reveals that the detector response decreases with increasing distance from the PMT photocathode and towards the periphery of the detector. This reduction can be attributed to issues with photon transmission to the photocathode of the PMT leading to losses and inhomogeneities during the transmission of the light. Finally, the response of the detector as a function of the working bias was also studied. A 2 MeV proton beam was positioned around the central region of the detector, and a voltage scan was performed from −850 V to −1100 V. Figure 9 (Top) shows the averaged temporal waveforms for each applied bias. From Figure 9, it can be observed that the waveforms exhibit a flat behavior with an initial hump, which is common to all the analyzed waveforms.

It is important to note that, rather than the applied voltage, the key factor here is the saturation of the PMT when the output signal exceeds 250 mV, as the signal for a given applied voltage depends on the type of incident particle. In this particular case, saturation occurs for voltage values exceeding 1050 V. When the flat-top level values are plotted as a function of the applied voltage, it is found that the signal amplitude increases with the applied voltage to the PMT within the studied range. In the case of waveforms corresponding to voltages exceeding 1050 V, the flat-top level was measured at the beginning of the signal, just after the initial hump and before the loss of linearity. The data set was fitted to a linear function, showing excellent agreement (R^2^ = 0.997) with the fitting model, which demonstrates the linear response of the detector to the applied bias to the PMT, as illustrated in Figure 9 (Bottom).

### 3.3. Single Ion Detection

One of the main advantages of this new type of thin scintillator is its suitability for measuring single ions. Measuring single ions is crucial for advancing high-resolution analytical techniques, enabling precise characterization in fields such as mass spectrometry, nanotechnology, and fundamental particle research [40].

**Figure 10:**
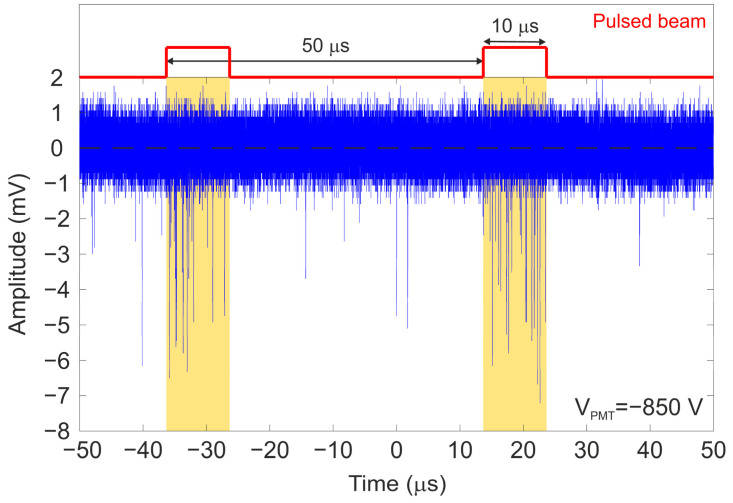
Temporal waveform measured with the detector irradiated with a 2 MeV pulsed proton beam. The PMT was polarized at −850 V and the pulsed beam has a frequency of 20 kHz and a width of 10 μs.

To verify this, the ion beam current of the 2 MeV proton beam was reduced to 5–10 pA; subsequently, the ion beam was pulsed with a pulse width of 10 μs and a frequency of 20 kHz, resulting in an ion rate of 500 protons/pulse over the scintillator. This ion rate is low enough to prevent pileup events and successfully measure single ions. To estimate the occurrence rate of the dark current of the PMT, two sets of measurements were performed under complete darkness and without the ion beam at two different bias voltages: −850 V and −1000 V. From the analysis of several waveforms, the dark current occurrences were estimated to be approximately 0.9 events/μs at −1000 V and approximately 0.2 events/μs at −850 V. As discussed previously, a major issue encountered was the discrepancy between the thickness provided by the supplier and the actual thickness measured by RBS. This discrepancy has been the main setback in this analysis, as the amplitude of the proton waveforms is similar to the amplitude of the dark current inherent to this PMT at the working bias of −850 V, which produces the minimum occurrences of dark current. If the thickness of the scintillator had matched the nominal value provided by the manufacturer, the energy deposited by the ion would have been significant enough to distinguish between signals generated by the ion beam and the dark current. Figure 10 illustrates an example of the temporal response of the detector biased to −850 V within a 100 μs time window.

In Figure 10, the region of 40 μs without the ion beam shows that dark current signals appear over the background distribution but much less frequently than in the regions where the ion beam hits the sample (indicated in the graph with yellow rectangles). This analysis highlights the detection of single ions, showing that during the periods when the beam hits the sample, the number of signals is significantly higher than the dark current occurrence rate at −850 V.

### 3.4. Angular Divergence: Transmission Through the Collimator Slits

In a focusing system utilizing a magnetostatic lens, the presence of a collimator slit is crucial to mitigate aberrations during the focusing process, particularly given the ion beam’s dimensions prior to entering the slit. However, the micrometric dimensions of the collimator slit aperture compromise the transmission of the ion beam to the vacuum chamber, which is particularly critical for systems operating in transmission mode. Therefore, one of the fundamental parameters for characterizing the system is the estimation of beam transmission through the collimator slits.

In our experimental setup, the slits are located at the entrance of the nuclear microprobe, in front of the scanning system and behind the plastic detector, which is positioned approximately 38 cm upstream of the slits. They consist of a set of cross X–Y slits that were adjusted to create a square aperture set to 0.1 × 0.1 cm^2^. It is important to highlight that the placement of the plastic detector prior to the focusing section is essential; positioning the detector downstream of this section would induce angular and energy straggling of the ion beam as it passes through the scintillator, consequently compromising the beam’s focusing conditions. Thus, not all incident ions will pass through the slits, as their angular deviation may exceed the field of view (FOV) of the slits as measured from the plastic detector. For our setup, the FOV is given by arctan (0.05/38) ≈ 0.075°. The number of ions transmitted through the collimator slits was determined via SRIM simulations, using the actual layout (composition and thickness) described in a previous section. Simulations were conducted using 2 MeV and 3 MeV proton beams (1 × 10^5^ ions per simulation). SRIM’s transmission mode allows us to obtain the coordinates and direction cosines of the ions that reach the outer surface of the plastic detector and transmit through it. From the SRIM simulations, the transmitted energy distributions were obtained, resulting in an average energy loss of approximately 370 keV for the 2 MeV proton beam and approximately 260 keV for the 3 MeV proton beam. If we assume that, once it exits the plastic detector, each ion follows a straight path while traveling through a vacuum, the equation of the line that defines the trajectory of each ion can be obtained. To derive the parametric equation of a line passing through a point P(x_0_,y_0_,z_0_) and having a direction given by a unit vector with components corresponding to the direction cosines (l,m,n), the parametric equation of the line can be expressed as Equation (2):

(2) $$\vec{r}\left(\alpha \right)=(x\left(\alpha \right),y\left(\alpha \right),z(\alpha ))=\left\{\;\begin{array}{c} x(\alpha )=x_{0}+\alpha \cdot l\\ y\left(\alpha \right)=y_{0}+\alpha \cdot m\\ z(\alpha )=z_{0}+\alpha \cdot n \end{array}\right.$$

**Figure 11:**
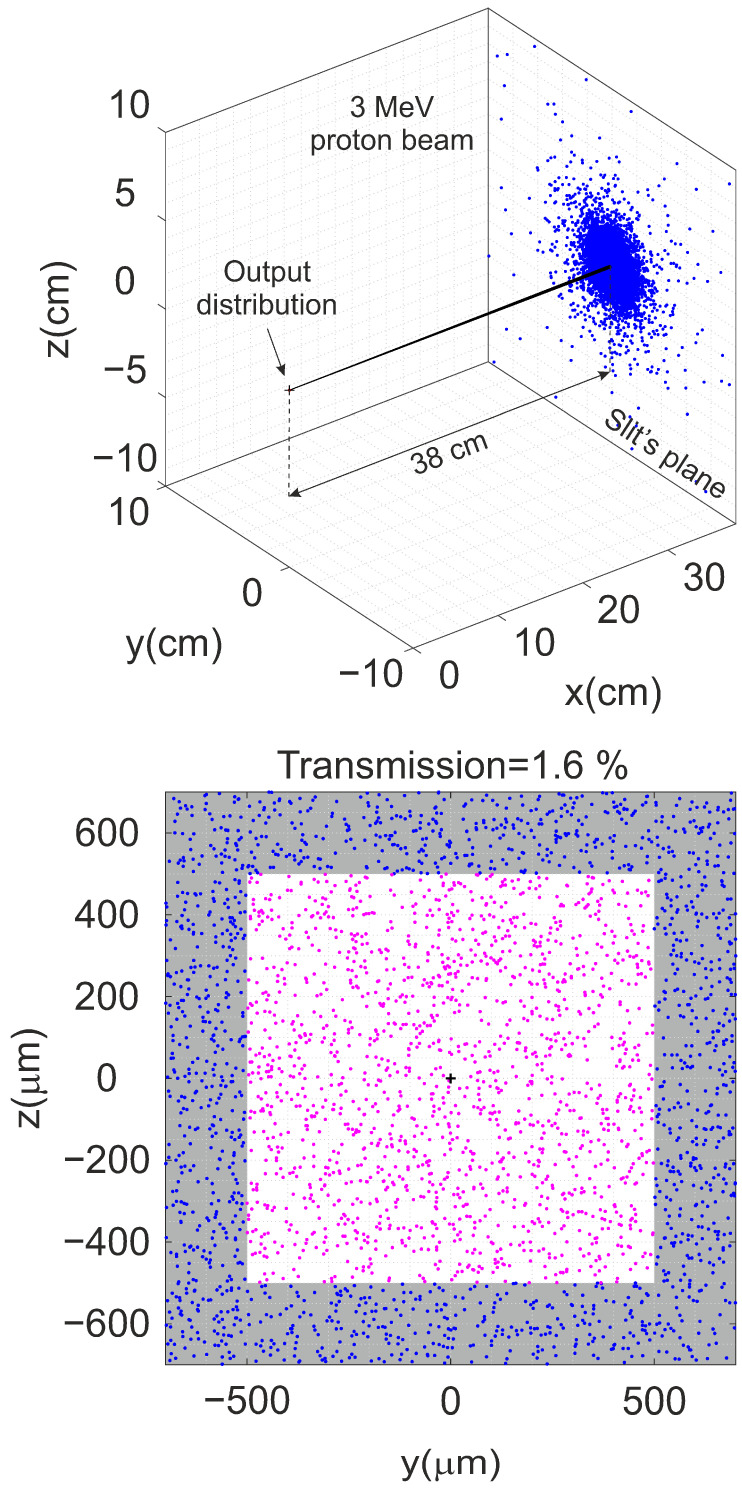
(**Top**) Simulated distribution of 1 × 10^5^ ions reaching the collimator slit plane for a 3 MeV proton beam. The black lines indicate the trajectories of the ions that transmit through the slits. (**Bottom**) Zoomed area of 1400 × 1400 μm^2^ showing the aperture of the slits (1000 × 1000 μm^2^). The magenta points represent the ions that pass through the slits.

Figure 11 (Top) shows the ion distribution (blue points) over the slit plane obtained using this method for a 3 MeV proton beam, starting from the initial distribution at the outer surface of the plastic detector provided by SRIM. The black lines indicate the trajectories of the ions that pass through the collimator slits (not visible in the figure due to the difference in scale between the slit dimensions and the final ion distribution at the slit position).

As expected, the simulations showed that the beam distribution spreads more for 2 MeV protons, where the angular scattering is greater. Moreover, almost all ions are located within circular-shaped regions around the incidence direction, with only a small number scattering at large angles. Figure 11 (Bottom) shows a zoomed-in view of the ion distribution around the aperture of the slits. The magenta points represent the ions that transmit through the slits along their trajectories, while the ions shown in blue collide with the slits. For each energy, the transmission was calculated as the ratio of the transmitted ions with respect to the total number of simulated ions. Considering the dimensions of the aperture, less than 0.6% of the incident ions will transmit through the collimator slits for the 2 MeV proton beam. This percentage increases to around 1.6% for the 3 MeV proton beam. It is important to remark that the experimental transmission values are indeed lower than those obtained through SRIM. One of the limitations of the simulations conducted is that they assume all ions impinge normally on the detector and that the beam is point-like. In reality, the plastic detector is positioned before the collimator slits, so the physical dimensions and divergence of the incident ion beam differ from those considered in the SRIM simulations. Therefore, the simulated value can be regarded as an upper limit, representing an ideal case scenario.

These results highlight the importance of using a plastic detector instead of a thin semiconductor detector before the collimator slits, as the limitations imposed by the slits require a high ion current (on the order of a few kHz) at the input, which can disable or damage any semiconductor detector used in transmission mode.

### 3.5. Time of Flight and Temporal Resolution

**Figure 12:**
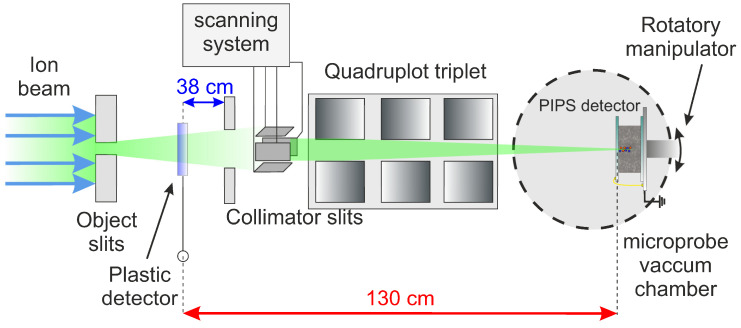
Schematic of the ToF system at the CNA nuclear microprobe, showing detectors positioned 130 cm apart. The plastic detector was installed 38 cm before the collimator slits.

The ToF technique is a versatile and precise method for measuring the travel time of particles between two detection systems [41]. The basic setup of a ToF system consists of two primary detectors aligned along a straight path: the Start detector and the Stop detector, separated by a known distance. In our experimental setup, the Start detector (plastic detector) is positioned before the collimator slits, as described in previous sections. When an ion passes through the Start detector, it generates a start signal that marks the precise moment of the particle interaction. An ion-implanted Silicon detector supplied by ORTEC, with an active area of 25 mm^2^, was installed in the ion beam microbeam chamber as the Stop detector. The detector was mounted on a rotary manipulator, allowing it to be positioned both on-axis and off-axis relative to the ion beam to prevent radiation damage during ion beam optimization. The distance between the two detectors was fixed at 130 cm. Initially, the homogeneity of the ORTEC detector response was tested using the Ion Beam Induced Charge technique (IBIC) [42] with a 3 MeV focused proton beam before measuring the ToF distribution. The IBIC analysis revealed high homogeneity in the detector response, ensuring no dependence on the impact point. An overview of the experimental configuration is shown in Figure 12.

**Figure 13:**
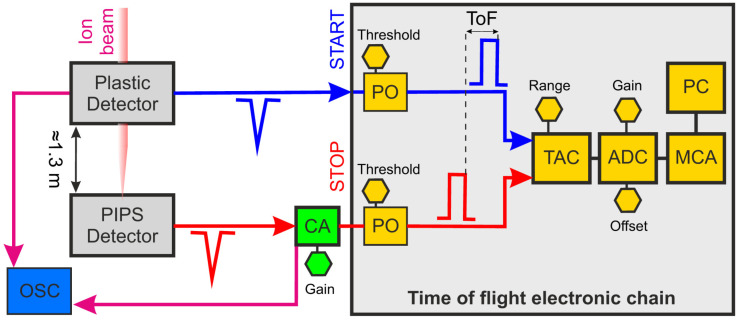
Electronic chain diagram for ToF experiments. The components are labeled as follows: CA (Current Amplifier), PO (Pick-off), TAC (Time-to-Amplitude Converter), ADC (Analog-to-Digital Converter), MCA (Multi-Channel Analyzer), and OSC (Oscilloscope).

By setting the ionic species and energy, the ToF distribution (to precisely measure the difference of the arrival times between the two signals generated in one event) of the ion beam can be obtained using a standard electronic chain for timing measurements [43]. The typical block diagram of the modules used in a ToF measurement includes the following main elements: a set of pico-timing discriminators (pick-offs), a Time-to-Amplitude Converter (TAC), an Analog-to-Digital Converter (ADC), and a Multi-Channel Analyzer (MCA). Additionally, raw signals from both detectors may be processed with a fast oscilloscope (OSC). A schematic of the electronic chain for timing measurement is displayed in Figure 13.

**Figure 14:**
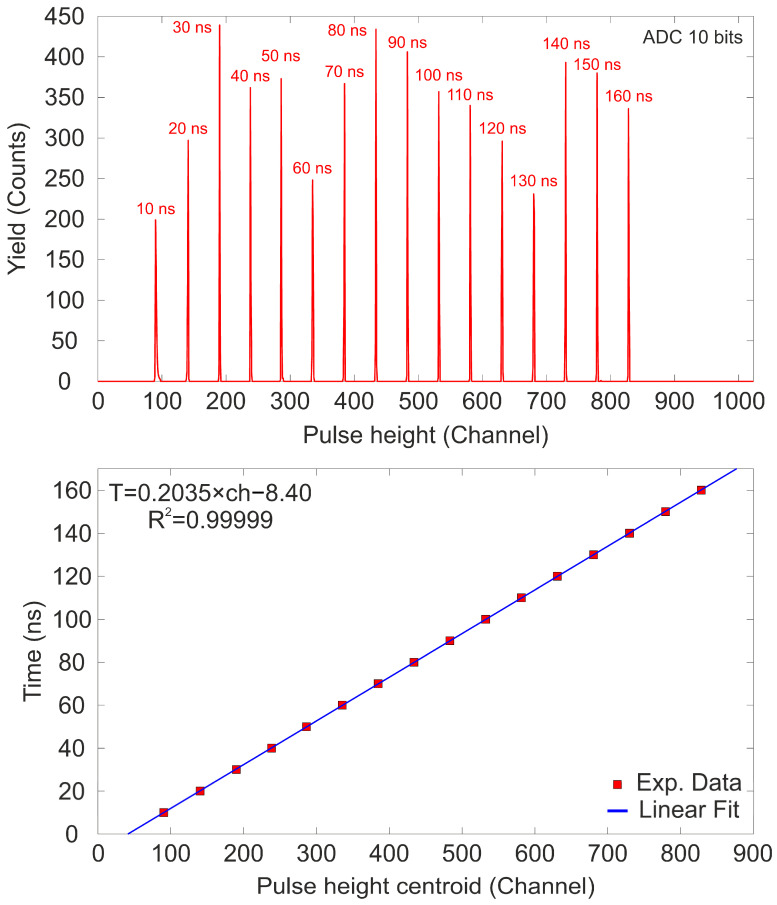
(**Top**) Pulse height spectrum obtained with a TC set to a period of 10 ns and a range of 160 ns. The TAC was set to a range of 200 ns. (**Bottom**) Time periods plotted against the most probable centroid of the peaks, with the blue line representing the best linear fit.

With this configuration, the output signal from the plastic detector (Start signal) following the passage of an ion and the signal from the Si detector when it detects the same ion (Stop signal) are transmitted to the set of pick-offs (Model 9307 by Ortec, Oak Ridge, TN, USA). They are typically used in applications with ultra-fast detectors requiring picosecond precision, and ultra-fast circuits are incorporated to minimize time slewing [44]. The output signals from the plastic detector do not need any additional amplification; however, in the case of the PIPS detector, the output signal was amplified using a C2-HV Broadband Amplifier (Cividec, Vienna, Austria) with a gain of +40 dB and an input/output impedance of 50 Ω [45]. After processing each pulse to shape the signals appropriately, both signals are sent to the TAC (Model 567 by Ametek, Oak Ridge, TN, USA). Finally, the output pulse is digitized by an ADC (OM-1000e from Oxford Microbeams, Bicester, UK) and the amplitude distribution is processed by an MCA. After completing the signal processing, a histogram of the time elapsed between two detected events at the Start and Stop detectors, known as the time spectrum, can be obtained. The initial step in ensuring accurate ToF measurement involves the calibration of the electronic chain to correctly assign the correct time intervals to the output signals from the MCA [46]. The calibration of the electronic chain was conducted using a Time Calibrator (TC) module (Model 462 by Ortec), which generates two output signals, for each trigger event, of known separation in time delays that mimic the Start and Stop signals. For the calibration, the TC was set to produce a signal train with a 10 ns period and a range of 160 ns. The time difference between the TC signals for each event is triggered by a random generator with the same probability distribution [47]. The TAC was set to a range of 200 ns, so it only processes signals with time differences of 200 ns or less, generating an analog pulse with an amplitude ranging from 0 V to 10 V, proportional to the time difference between the Start and Stop signals. Figure 14 (Top) shows the pulse height spectrum obtained during the calibration, where a set of 16 peaks separated by the TC set period are clearly visible.

**Figure 15:**
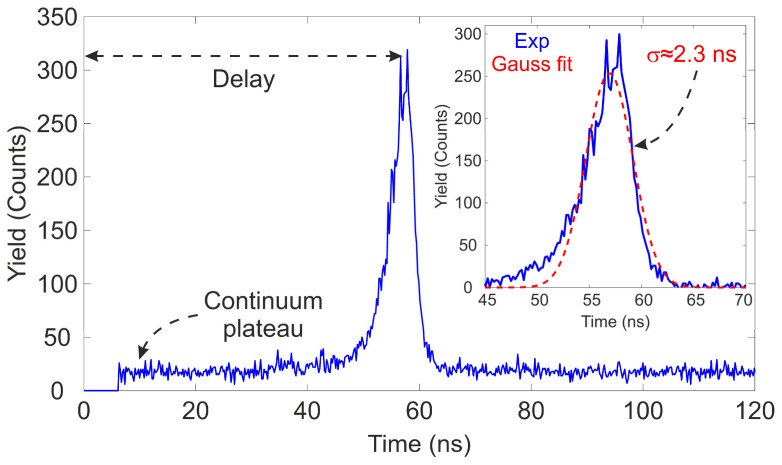
ToF spectrum of a 3 MeV proton beam traveling 130 cm. The inset shows a zoom of the main peak with the best Gaussian fit, indicating a standard deviation σ = 2.3 ns.

The length of the coaxial cables used for connections introduces an additional temporal delay (approximately 5 ns/m for 50 Ω coaxial cables [48]) between the two signals. However, this delay is constant and does not significantly affect the system’s temporal resolution, as the cable lengths for both signals were kept the same and short enough to avoid distorting the pulse shapes of high-frequency signals [49]. The MCA was configured with a resolution of 10 bits (1024 channels). Figure 14 (Bottom) depicts the most probable centroid position (obtained from Gaussian fits of the peaks in the pulse height spectrum) of each peak as a function of the TC periods (red squares). The blue line represents the best fit to the data obtained through least squares fitting. It is observed that, within the selected range, the system exhibits linear behavior (R^2^ = 0.99999), and the calibration enables the establishment of a channel–time interval relationship, essential for ToF measurements and the determination of the temporal resolution. The performance of the calibrated setup was tested by measuring the ToF distribution of a 3 MeV proton beam using the same electronic chain. For this measurement, the plastic detector was fully inserted and biased at −1150 V, the PIPS detector was exposed to the ion beam with a working bias of +70 V, and the count rate at the Si detector was reduced to a few particles per second. The voltage for the PMT was selected because the pick-off modules require a minimum threshold in the amplitude of the input waveforms to process the signal. Due to the angular divergence and transmission of the ion beam to the microprobe chamber, as well as the appearance of uncorrelated events due to the dark current in the PMT (which frequency depends on the applied bias), the count rate is higher in the plastic detector compared to the PIPS detector. To discriminate false coincidence events and minimize dead time in the TAC, the start signal was set to the pulse with the lower count rate, which, in this case, corresponds to the PIPS detector. The TAC range was set to 200 ns, and the threshold discriminator levels for the pick-offs were adjusted to be above the electronic noise level. The beam, after passing through the quadrupole triplet, was focused to a size of 2–5 μm. Figure 15 displays the ToF spectrum measured for a 3 MeV proton beam.

Two notable features are evident from this figure: The main peak represents the temporal distribution of protons (true coincidence events) traveling and detected in both detectors. Additionally, the pulse height spectra are observed to lie atop a continuous plateau, which is attributed to the random occurrence of the dark current, resulting in uncorrelated time events. Even though the main peak is not completely symmetric, the peak centroid and temporal resolution of the system are determined from the mean value and standard deviation of the Gaussian fit to the main peak. From the inner figure, it can be observed that there is good agreement between the temporal spectrum peak (continuous blue line) and the Gaussian fit (dashed red line). From this fitting, it is found that the most probable ToF for the proton beam is ≈56.9 ns, with a standard deviation of 2.3 ns. This average ToF perfectly matches the value obtained through SRIM simulations, using the relativistic velocity equation and the projection along the incoming direction. For ToF measurements, the definition of time resolution varies among different authors. In some works, it is conventionally given by the root mean square (RMS) of the timing difference between the start and stop signals [50]. However, the common consensus defines the time resolution of the acquisition system as the full width at half maximum (FWHM) of a prompt coincidence time distribution, assuming that the timing uncertainties in both branches are Gaussian type [51]. In our experimental setup,

(3) $$FWHM=2\sqrt{2\ln(2)}\sigma \approx 5.4\;ns$$

It is important to note that this time resolution does not solely reflect the resolution of the plastic detector. Considering negligible contributions from other sources (such as time-walk, readout electronics, time jitter, Landau fluctuations, distortions, etc. [52]) to the temporal resolution, the measured resolution can be expressed as the quadratic sum of the resolutions of the involved detectors as follows:

(4) $$\sigma_{Measured}^{2}\approx \sigma_{Plastic}^{2}+\sigma_{PIPS}^{2}$$

where σ_Measured_, σ_Plastic_, and σ_PIPS_ correspond to the measured temporal resolution, the temporal resolution of the plastic detector, and the temporal resolution of the PIPS detector, respectively. It should be noted that the temporal resolution of the PIPS detector is not negligible compared to that of the plastic detector, so the measured temporal resolution represents an upper limit of the actual resolution.

**Figure 16:**
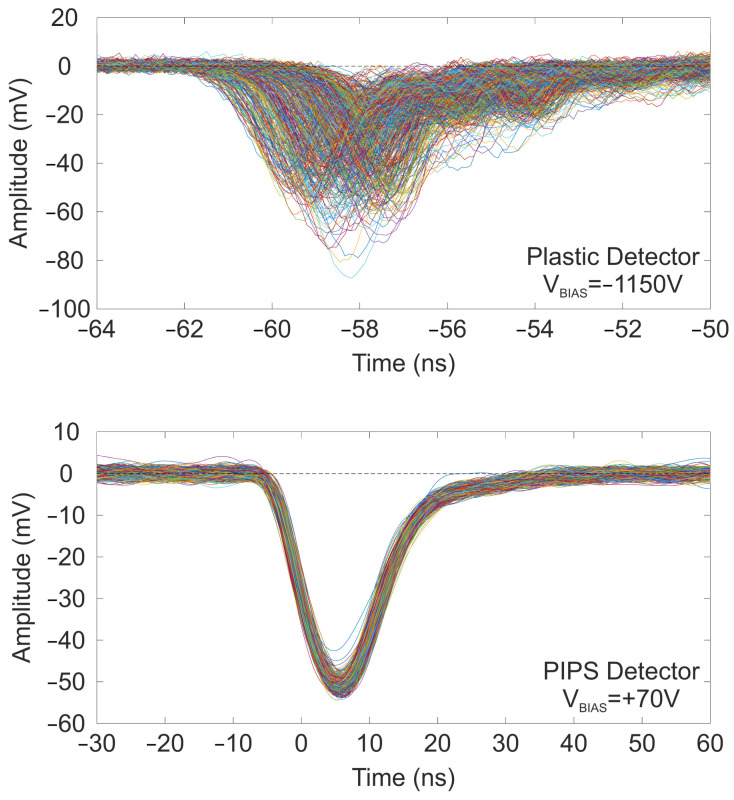
Selected waveforms for the plastic detector (**Top**) and the PIPS detector (**Bottom**). The waveforms were recorded using the two-channel trigger mode on the oscilloscope.

In addition to the ToF measurement using the electronic chain, the proper functioning of the ToF setup was further verified by recording both the Start and Stop signals for each event using the LeCroy HDO9404 high-speed oscilloscope. A set of ≈1000 waveforms are shown in Figure 16 for the plastic detector (Top) and PIPS detector (Bottom).

To avoid situations where an ion does not hit the other detector, the oscilloscope was configured in a two-channel trigger mode, requiring both conditions to be met to indicate a coincidence event. An event is recorded only when the waveforms simultaneously reach trigger levels of −37.8 mV for the PIPS detector and −29.8 mV for the plastic detector. As shown in Figure 16, the individual waveforms from the plastic detector are faster (on the order of a few ns) compared to the signals from the PIPS detector (on the order of tens of ns), resulting in much narrower waveforms from the plastic detector than from the PIPS detector. However, the plastic scintillator has a much wider peak distribution than the PIPS detector due to the oscilloscope configuration. With this setup, the arrival time of the plastic detector waveforms is measured for each event that satisfies its own trigger conditions. However, the instant of time (arrival time) at which these events occur is marked by the PIPS detector, as this system defines the origin of time for the coupled system in our current configuration. As the time-of-flight of the ions follows a distribution due to energy straggling as they pass through the plastic detector, the collected waveforms present a wider distribution. To obtain the ToF as the difference in the arrival times of both signals, one would typically use time–pickoff methods such as Leading edge triggering, Fast zero-crossing triggering, or the Constant fraction discriminator [53]. However, this issue is far from the main goal of this measurement, which was to verify that the results obtained with the ToF electronics are consistent with those obtained by directly measuring the signals on the oscilloscope.

## 4. Conclusions

In this study, a detector based on a novel ultra-thin organic scintillator was successfully installed and tested at the CNA. This system represented the first implementation of an external trigger in the nuclear microprobe of this facility, demonstrating its potential for both single ion detection and ToF measurements. One significant finding was that the experimental thickness of the scintillator sheet was notably lower than the specified commercial thickness. This deviation posed challenges regarding signal amplitude, as observed during the evaluation of the dark current. However, it also contributed to reduced energy straggling, resulting in a less perturbed beam, which is critical for precise timing measurements. The detector response showed a strong dependence on the impact position of MeV protons; nevertheless, it remained predominantly linear with the applied working bias. Although this spatial dependency required consideration, it did not impede the system’s function as an external trigger in TRIBIC measurements, where the center of the beam was aligned with the center of the detector at the optimal position of the linear manipulator. Moreover, a key advantage of this setup was its low transmission value through the collimator slits. Considering the dimensions of the aperture, less than 0.6% of the incident ions transmitted through the collimator slits for the 2 MeV proton beam, which increased to approximately 1.6% for a 3 MeV proton beam. This low transmission underscores the need for high currents at the detector location, making the scintillator an optimal choice due to its superior radiation hardness, which enables operation under conditions that would be detrimental to thin semiconductor detectors used in transmission mode while still achieving acceptable transmission levels to the microprobe chamber. In conclusion, this new setup shows great potential and significantly enhances the capabilities of the ion beam nuclear microprobe. Future work will focus on replacing the PIPS detector with a Low Gain Avalanche Detector (LGAD), which offers negligible temporal resolution issues, thereby allowing for a direct measurement of the temporal resolution of the plastic scintillator.

## Figures and Tables

**Table 1:** Characteristics of the EJ-214.

Efficiency (photons/1 MeV e^−^)	9000
Maximum Emission (nm)	435
Decay Time (ns)	2
No. of H Atoms per cm^3^ (×10^22^)	5.18
No. of C Atoms per cm^3^ (10^22^)	4.67
No. of N Atoms per cm^3^ (10^19^)	4.89
No. of O Atoms per cm^3^ (10^19^)	2.59
Density (g/cm^3^)	1.02
Softening Point (°C)	60

**Table 2:** Aluminized mylar: SIMNRA results.

Structure	Aluminum	Mylar
Composition (%)	Al	H_37_C_45_O_18_
Thickness (10^15^ at/cm^2^)	490	23,900
Physical thickness	81 nm	2.39 μm

**Table 3:** Scintillator: SIMNRA results.

Structure	EJ-214
Composition (%)	H_52.5_C_47.42_O_0.05_N_0.03_
Thickness (10^15^ at/cm^2^)	140,000
Physical thickness	13–14 μm

## Data Availability

The original contributions presented in the study are included in the article; further inquiries can be directed to the corresponding author.
